# New horizons in cooking and mealtime interventions for mild cognitive impairment and early dementia: a scoping review

**DOI:** 10.1016/j.jnha.2026.101009

**Published:** 2026-10-20

**Authors:** Sarah M. Bannon, Renata Elizalde Carrillo, Sydney McCage, Muhammad Hamza Shahab, Grace O. Allison, Aida Aminpour, Carrie Levinson, Alexander M. Presciutti, Anne Fishel

**Affiliations:** a Brain Injury Research Center, Department of Rehabilitation Medicine and Human Performance, Icahn School of Medicine at Mount Sinai Hospital, New York, NY, United States of America; b Gustave L. and Janet W. Levy Library, Icahn School of Medicine at Mount Sinai Hospital, New York, NY, United States of America; c Department of Psychiatry, Massachusetts General Hospital, Boston, MA, United States of America

**Keywords:** Dementia, Cooking, Mealtime support, Behavioral interventions

## Abstract

**Background:**

Alzheimer’s disease and related dementias (ADRDs) are increasingly common conditions that are highly disruptive to individuals and families. ADRDs impact cognition and can undermine a person’s ability to participate in cooking, preparing meals, and engaging in conversations. Mealtime participation implicates key determinants of adequate nutritional intake and social connectedness. However, engaging in mealtime routines amid challenges can also exacerbate the stress and burden felt by family caregivers. Behavioral interventions can mitigate these downstream impacts, though they are currently underdeveloped.

**Objective:**

To summarize the literature on cooking and mealtime interventions for community-dwelling people with dementia and their caregivers.

**Design:**

Scoping review.

**Methods:**

We searched databases up to May 2025 for English-language publications that described qualitative and quantitative research surrounding interventions focused on cooking and mealtime interventions for individuals living with subjective cognitive decline, mild cognitive impairment, and ADRDs. At least two reviewers independently identified publications and extracted population characteristics, intervention components, and intervention outcomes. Included articles were published between 2007–2024.

**Results:**

Qualitative and mixed methods studies (n = 12) highlighted potential mechanisms for intervention, including cultivating routines for mealtimes (e.g., eating together, minimizing distractions, adaptive tools) and caregiver coping skills and training (e.g., distress tolerance, communication to emphasize respect/dignity, and social routines). Intervention studies (n = 10) included caregiver skills training, education and cognitive rehabilitation-integrated cooking. Interventions were generally feasible; however, few were evaluated in randomized trials, and none underwent efficacy testing.

**Conclusion:**

Cooking and mealtime interventions hold promise for addressing determinants of aging in ADRDs, though further randomized controlled efficacy trials are needed.

## 1 Introduction

ADRDs produce cognitive, sensory, and functional deficits that make instrumental activities of daily living more difficult including cooking and preparing meals [1], along with maintaining adequate nutritional intake and engaging in mealtime conversation [2,3]. The ability to independently prepare and consume meals is associated with better outcomes for older adults [4], yet is commonly disrupted by ADRDs. Mealtime routines have a critical influence on nutritional intake and comorbid challenges that frequently accompany ADRDs (e.g., weight loss, malnutrition, dehydration) [5]. Approximately 14 % of individuals living with ADRDs exhibit signs of malnutrition in the early period after ADRD diagnosis [6], which is a well-documented risk factor for symptom progression [6,7]. Further, many experience social withdrawal and isolation given their reduced ability to participate in daily tasks [8]. Like malnutrition, social isolation is also a robust predictor of ADRD progression [9] and can amplify malnutrition risk [10].

For people living with ADRDs, mealtime routine participation can play a protective role in promoting adequate nutritional intake and aging in place [5,11]. Herein, mealtime routine participation in earlier stages of dementia is defined as the extent to which an individual routinely engages in the behavioral, cognitive, and social components of eating occasions [3]. Examples include awareness of and orientation to mealtimes, initiating and sustaining eating behaviors, and interacting with the physical and social mealtime environment (e.g., setting the table, initiating and/or joining in conversation, helping prepare or clean surrounding meals). Support at home is important for routine mealtime participation given that the majority (∼70 %) of individuals with ADRDs reside at home [12], and roughly 60 % of those living with ADRDs at home are not able to independently complete aspects of mealtime routines (e.g., food shopping, cooking or preparing meals) [8]. As a result, caregivers (i.e., family, friends, or familiar persons providing routine support) take on a greater role in mealtime routines, leading to increased stress and burden [5]. Over time, the changes in roles and mealtime routines can heighten distress surrounding mealtimes, strain relationships, and undermine the quality of care received from caregivers [6,7].

Shared mealtimes also play an important part in shaping family traditions and identities, and provide family members with a sense of belonging, comfort, stress relief, and familiarity [13,14]. The recognition of the importance of mealtimes for family health and wellbeing has led to the development of cooking and mealtime interventions, which are defined herein as programs that provide education and skills practice to address challenges related to meal preparation and cooking, eating behavior, and social engagement and connectedness [4,5,15]. Unfortunately, most interventions are focused on the advanced stages of ADRDs, and few resources prioritize early periods in the disease progression [11].

Our team’s broader goal was to provide an overview of programs that could simultaneously: (1) improve nutritional intake and physical wellbeing as well as the social engagement and emotional health of individuals living with dementia, and (2) reduce uncertainty, overwhelm, burden, and isolation among caregivers. Interventions can be used to restore the normalcy of important mealtime rituals, ease role shifts, and promote positive adjustment to ADRDs. Analyzing existing literature would then inform the future development, testing, and implementation of cooking and mealtime interventions for community-dwelling people living with ADRDs and their caregivers. We examined both the qualitative and quantitative literature surrounding cooking and mealtime programs for individuals with cognitive decline, mild cognitive impairment, or dementia and their caregivers. We focused on the following questions:

1 What are the perceived challenges, coping strategies and potential interventions, and preferred outcomes of individuals and caregivers navigating mild cognitive impairment and ADRDs surrounding mealtimes?
2 What are the intervention characteristics (procedures, format, content) and reported benefits of existing mealtime interventions for individuals and caregivers navigating mild cognitive impairment and ADRDs?

## 2 Methods

We adhered to the PRISMA-SCR guidelines [16] for scoping reviews and registered the review protocol on OSF (osf.io/guezx). Our review was limited to articles describing cooking or mealtime experiences for individuals and caregivers navigating dementia-related challenges. We included articles related to subjective cognitive decline, mild cognitive impairment, and ADRDs; articles written in English; and those involving individuals living with ADRDs and/or family caregivers that were community-dwelling. We included both qualitative and quantitative literature, including observational studies, case studies, open pilot trials, controlled trials, and unpublished theses or dissertations consistent with scoping review guidelines [5,16,17]. We excluded articles that focused on formal or professional caregivers and those characterizing long term or residential care contexts (e.g., long term care, memory care facilities, and/or skilled nursing facilities). We also excluded systematic reviews, commentaries, and opinion papers. Articles were retrieved from four databases (Embase (Ovid), MEDLINE (Ovid), PsycINFO (Ovid), CINAHL) with relevant key words through May 2025 (see Supplemental Table S1). To ensure a comprehensive analysis, our search strategy included all relevant articles from inception through May 9, 2025.

We performed screening for eligibility using Covidence [18]. All databases were searched, and articles were identified, with duplicates removed in Covidence. At least two members of the study team (SB, MHS, GA, SM, MP, AA) screened titles and abstracts based on the eligibility criteria. We resolved disagreements through consensus during weekly reviewer team meetings. Next, we reviewed potentially eligible articles through full-text review using the same process applied during title and abstract screening.

Importantly, we abstracted data using a data abstraction form developed by the review team (SB, SM, REC, MHS, AA) prior to full extraction (see Supplemental Table S2). The charting process captured key information from each source, including study design, population characteristics, recruitment procedures, analytic approaches, outcomes, and qualitative as well as quantitative results relevant to the review questions. The data extraction tool was revised and refined throughout the process to ensure clarity and comprehensiveness. Lastly, and following the method as above, data were charted independently, and any discrepancies were resolved through team discussion to reach consensus.

Given the nascency of research in this area, we extracted data from included articles to provide an overview of the existing literature. This included information on overarching themes of qualitative studies focused on cooking and mealtime experiences, intervention characteristics (procedures, format, content), target outcomes (e.g., quality of care received, social connectedness, well-being, relationship satisfaction), qualitative descriptions of perceived benefits for individuals and caregivers and hypothesized mechanisms of intervention. Consistent with PRISMA-ScR guidelines, we did not conduct critical appraisals of individual sources of evidence.

We engaged in categorization and narrative synthesis of results. Summarized findings for included studies were compiled in a tabular format by the review team using the data elements aligned with our primary research questions. We revised our table of extracted findings to promote clarity and conciseness during team discussions.

## 3 Results

### 3.1 Study selection

Fig. 1 presents a flow diagram of included articles. Our search of databases identified a total of 1,513 articles. After removing duplicates, 922 articles remained for screening. We conducted a two-stage screening process, first screening using titles and abstracts, and then screening using full texts. Overall, we excluded 817 articles during the title and abstract screening stage, and 82 additional articles during full text screening. We included a total of 22 studies in this scoping review.

**Fig. 1:**
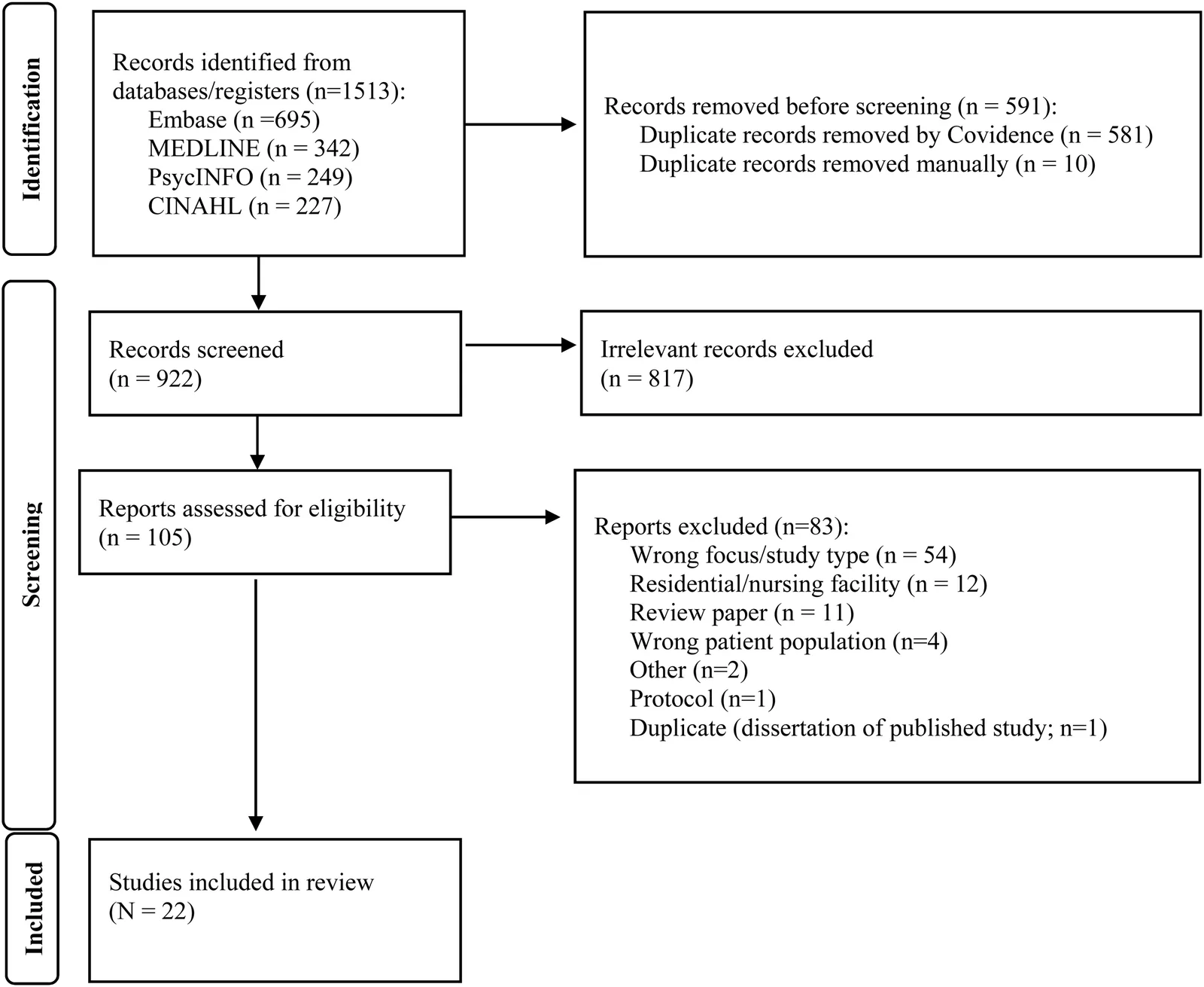
PRISMA flow diagram of included articles (N = 22).

### 3.2 Characteristics of included studies

Supplemental Table S3 provides a description of characteristics of the 22 included studies. Studies were published between 2007 and 2024 and conducted in 9 countries: Canada (n = 5), United States (n = 4), United Kingdom (n = 4), Sweden (n = 3), Japan (n = 2), Australia (n = 1), Brazil (n = 1), Hong Kong (n = 1), and Malaysia (n = 1). Included studies were peer reviewed publications (n = 21) and an unpublished dissertation study (n = 1). Studies included qualitative or mixed methods designs (n = 12) as well as randomized or non-randomized intervention trials (n = 10). Persons living with dementia (n = 13), caregivers (n = 16) and healthcare professionals (n = 3) were included in studies as participants. Most studies recruited from community settings (n = 16), while others recruited participants from clinic settings (n = 9), with some recruiting from a combination of community, clinic, and residential settings.

### 3.3 Perceived challenges, coping strategies, and potential intervention components

Table 1 provides a detailed summary of all findings from qualitative and mixed methods studies. Studies explored the perceptions of people living with dementia, family caregivers, and healthcare professionals. Findings are organized in terms of perceived challenges, coping strategies and potential interventions, and preferred outcomes of interventions targeting mealtime routines.

**Table 1:** Summary of findings from qualitative/mixed methods and quantitative prospective studies (n = 12).

Reference	Study Aim	Study Design/ Participant Characteristics	Relevant Findings	
			Cooking/Mealtime Challenges	Intervention Recommendations
1. Ball et al. 2015	To explore the perceptions of family carers of people with dementia, with emphasis on feeding-related challenges, burden of care attributable to nutrition support, and practical strategies developed to address these challenges	Descriptive exploratory study using semi structured interviews; synthesized findings using thematic analysis **Caregivers (N = 14)**: Age: 58 +- 17.8 years Time since care recipient’s dementia diagnosis: 1–14 years Sex: Female (n = 12) Male (n = 2) Relationship to care recipient: Husbands (n = 2) Wife (n = 4) Daughter (n = 8)	Cognitive (most common): -forgetfulness - executive dysfunction Emotional: -social isolation -reluctance to be fed by caregivers Practical/ functional challenges: -difficulty using cutlery -visuospatial problems	Caregiver-focused: - Distress tolerance/ cultivating patience -Frequent gentle and encouraging communication to eat -Accommodating taste preferences Mealtime-focused: -Eating together as a family -Allowing ample time for meals -Encouraging social interaction -Minimizing distractions -Maintaining mealtime routines -Improving food presentation (e.g., cutting into small pieces, using finger foods) Address feeding challenges: -Improving food intake -Minimizing or preventing weight loss -Reduce care partner anxiety due to wasting food
2. Fjellström et al., 2010	To examine how people living with persons with Alzheimer's disease perceived everyday life aspects of food choices, cooking, and food-related work.	Qualitative study of caregivers; data collected via focus groups and analyzed with thematic analysis **Caregivers (N = 17):** Sex: Female (n = 11) Male (n = 6) Time since care recipient’s dementia diagnosis: M = 2 years (Range: 0.5–4)	Emotional challenges: -insecurity about cooking abilities how to prepare nutritional meals -higher strain and burden from increased tasks Practical challenges: -shift from sharing food duties to caregivers handling tasks alone -inexperienced men caregivers struggled more -weight-management challenges for underweight care recipients -lack of clear nutritional guidance	N/A
3. Genoe et al., 2012	To examine how persons with dementia and their caregivers respond to mealtime changes that occur throughout the dementia journey.	Qualitative analysis of interviews using grounded theory approach to synthesis of findings **Persons living with dementia (n = 27):** Age: 56–88 Sex: Female (n = 16) Male (n = 11) Diagnosis: Frontal-temporal lobe dementia (n = 1) Vascular dementia (n = 1) Alzheimer's disease (n = 25) **Caregivers (N = 28):** Age: 30–88 Sex: Female (n = 16) Male (n = 12) Relationship: Spouse (n = 19) Child (n = 8)	Resistance: -people with dementia resist change to preserve identity and dependence -resistance can be a protective and limiting factor Holding patterns: -family members delay or avoid implementing changes to reduce overwhelm and maintain dignity	Acceptance and adaptation: -family members focus on continuous adaptation to cope with challenges, less likely to cultivate emotional acceptance Resilience and personal growth: -focus on finding meaning and focusing on present joys - cultivating responsiveness to changes as dementia progresses Caregiver readiness: -caregivers take the lead in managing and easing transitions for person living with dementia
4. Johannson et al., 2017	To explore and describe staff views on how to improve mealtimes for persons with dementia who are still living at home.	Qualitative study using four focus group interviews with staff **Caregivers (N = 22)**: Staff working close to persons with dementia, particularly those staff dealing with nutritional status and mealtimes. Position: Nursing assistant (n = 11) Nurse (n = 2) District nurse (n = 5) Social worker (n = 2) Occupational therapist (n = 2)	N/A	Individualized Support - meals should be familiar and tailored to personal history, preferences, and routines -support must be structured but flexible, allowing for the preservation of independence and dignity - knowing individual and their home situation crucial for providing effective meal-related support. Knowledge-Based Planning - systematic assessment of the person’s needs, including medical issues (e.g., medications, weight loss), family and social support, personal resources and existing abilities. - involvement of the partner/family as caregivers and recipients of support. - importance of ongoing evaluation and adjustment of care Meals at Home - maintain person’s role in meal preparation key to promoting self-worth and engagement. - assign simple, familiar tasks (e.g., peeling potatoes), supervise without taking over, using assistive devices (e.g., timers) - partner support and education (e.g., cooking classes) recommended to empower caregivers Meal Preparation - staff may need to take full responsibility for meals (e.g., using meals-on-wheels), should be done sensitively, especially when the person has a strong identity tied to cooking - gradual transition (e.g., starting with weekends only) can help maintain dignity Outside home - Eating at restaurants/ day programs a positive alternative to reduce stress on partners, improve appetite, routine, and social interaction
5. Juckett et al., 2024	To characterize barriers to participate in mealtime activities by people living with dementia. Also, to identify potential caregiver-led strategies to enhance mealtime activities.	**Health Professionals (N = 20):** Sex: Female (n = 17) Male (n = 3) Professional background: Occupational Therapist (n = 5) Nutrition program provider (n = 3) Registered Nurse (n = 3) Caseworker/ counselor (n = 3) Speech language pathologist (n = 2) Social worker (n = 2) Registered dietitian nutritionist (n = 2)	Cognitive challenges: -memory loss -attention issues -difficulty recognizing food/ identifying spoilage Functional challenges: - poor coordination, mobility, and posture -difficulty with cutlery /packaging -swallowing or dental problems increasing aspiration risk Practical challenges: - irregular eating patterns -changing food preferences and mealtimes -insufficient supervision -lack of training and resources on mealtime assistance and nutrition for people with dementia Environmental challenges: - cluttered spaces -lighting and appliance safety concerns -food storage concerns	Cognitive interventions: -visual/ written cues -timers or checklists -minimize distractions Functional interventions: -safety assessment -adaptive kitchen tools -plan for finger foods -supportive seating -color contrast dishware Practical interventions: -accessible education (flyers, instructional videos, hotlines on meal preparation, nutrition, and safe feeding practices) -community meal programs for delivery, set up, safety monitoring -declutter space, ensure lighting and appliance accessibility, monitor food storage
6. Keller et al., 2007	To explore the following: (1) the range of eating conduct changes of persons with dementia as described by their primary caregivers, (2) the strategies primary caregivers employ to cope with these changes, and (3) the issues or concerns that informal caregivers face with the mealtime experience.	Qualitative thematic analysis of interviews with family caregivers **Caregivers (N = 23):** Age: Mean = 65, (32–80) Sex: Female (n = 15) Male (n = 8) Time since care recipient’s diagnosis: 1−8 years	Caregiver strain and distress: -need to simplify meals to reduce strain -challenges navigating changing eating abilities and preferences -unpredictability as a source of fatigue -uncertainty about how and when to offer support Changes in eating abilities and preferences: -described “the shrinking plate” as dementia progressed, feeding constraints reduced food intake -declines in appetite, ability, or interest made meals smaller and more limited	Promoting social routines through mealtimes: -meaningful opportunities for conversation -often considered high point of the day for caregivers and recipients Mealtime enhancement: -goal of preserving routines, reducing challenges and uncertainty, and making experience more pleasurable for all involved
7. Keller et al., 2010	Focuses on three ways in which “Being Connected” occurs at mealtimes: being face to face, participating psychologically, and getting and giving support.	Qualitative grounded theory analysis of interviews of persons living with dementia and caregivers **Persons living with dementia (n = 27):** Age: 56−88 years Sex: Female (n = 16) Male (n = 11) **Caregivers: Mean Age; etc (n = 28):** Age: 30−88 years Sex: Female (n = 16) Male (n = 12) Relationships: Spouses (n = 19) Parent-child (n = 8)	N/A	Being connected at mealtimes: -being face-to-face and shared participation in mealtime tasks -cognitive and emotional engagement during meals -giving and receiving support while eating together Positive dyadic interactions: -mealtimes as avenue to have positive interactions that might be missing in other parts of daily live
8. Keller et al., 2015	To understand the meaning of mealtimes for persons with dementia and their family caregivers. Also, to describe the strategies devised and sued by these families living with dementia.	Longitudinal cohort study using qualitative grounded theory analysis and a constant comparative method. Interviewed persons living with dementia and caregivers together and separately. **Persons with dementia (n = 27):** Age: 56−88 Sex: Y1: Female (n = 16), male (n = 11) Y2: Female (n = 13), male (n = 10) Y3: Female (n = 11), male (n = 7) **Caregivers (n = 28):** Age: 30–88 Sex: Y1: Female (n = 16), male (n = 12) Y2: Female (n = 13), male (n = 10) Y3: Female (n = 10), male (n = 8)	N/A	Being connected: -taking time and focusing attention -communicating activities and staying informed -gaining knowledge, sharing and creating stories Honoring identity: -making shared decisions -providing support -taking part, enabling, and negotiating roles Adapting to an evolving life: -being creative -acceptance and understanding reality -promoting routines and traditions
9. Lam & Keller, 2015	To examine a specific ethnocultural group with cultural food-ways and caring expectations, to determine if the concept of honouring identity needed to be modified or extended.	Qualitative thematic analysis of interviews of Chinese Canadian caregivers **Persons with dementia: (n = 6)** Age: 80−100 Sex: Female n = 5 Male n = 5 **Caregivers (n = 8):** Sex: Female (n = 8)	N/A	Protecting dignity and identity: -providing culturally appropriate care in the midst of competing work/family demands -engaging in meaningful meal-related roles -routines and meal structure (e.g., soup at dinner) -importance of transferring culture across generations
10. Mole et al., 2021	To explore family caregiver perceptions and experiences with nutritional care of loved one with dementia living at home.	Qualitative thematic analysis of caregiver interviews **Caregivers (N = 8)** Mean Age: 69.6 Sex: Female n = 3 Male n = 5	Adaptations to caregiving role: -Changes in food provision, diet, routine, roles, and experience -Focus on approaching things like food shopping in “task based” way to “get things done quickly” -Changing roles and relationship with caregiver now “head chef” taking full control Emotional eating: -muddling through mealtimes and navigating food guilt -caregivers tasked with managing intake of food, and especially alcohol -desire to balance healthy diet with lightness and fun at mealtimes	N/A
11. Papachristou et al., 2013	To explore the impact of dementia progression on food-related processes from the perspectives of informal caregivers. In addition, the aim of the manuscript was to document the methodology used and to disseminate the findings to researchers, care providers, and policy makers.	Qualitative thematic analysis of semi structured interviews of caregivers **Caregivers (N = 20)** Sex: Female n = 10 Male n = 10 Relationship: Spouse n = 2 Child n = 4 Friend n = 1 Sister n = 2	Food shopping challenges: -rapid decline in shopping ability due to memory loss, navigation difficulties and wandering, trouble managing money Food preparation challenges: -kitchen safety concerns given gradual decline in ability to prepare food -shift to simple tasks (e.g., setting the table) Feeding challenges: -eating abilities declined last among food-related tasks -most kept a similar diet as before onset of dementia	N/A
12. Sharma et al., 2024	To shed light on the specific burden experienced by caregivers in Malaysian families, emphasizing the need for targeted interventions and support services to address their distinct needs.	Cross sectional mixed-methods study of caregivers **Caregivers (N = 31)**: Age: 31–50 Sex: Female n = 28 Male n = 3	Quantitative findings: -Most (90%) of people with dementia needed meal assistance -Most common issues: appetite changes (23%), food preferences (17%), and long mealtimes (44%) -Over 55% of caregivers felt burdened, with 52% reporting very high stress. -Causes of stress: behavioral symptoms (42%), lack of knowledge (21%), and high dependence (18%) -dementia severity and caregiver burden showed a moderate positive correlation (**r = 0.45**)	Quantitative findings: −77% used coping strategies like seeking help (61%) and taking breaks (52%) Coping strategies: -many relied on faith-based coping strategies such as praying, reading the Quran, singing religious songs, or cleaning to manage stress Unmet needs: - practical assistance, emotional support, and better information and training on dementia care

### 3.4 Perceived challenges

For persons living with ADRDs, functional challenges stemmed from cognitive symptoms (e.g., forgetfulness, memory loss, attention issues, and executive dysfunction), including difficulties recognizing food or signs of food spoilage while shopping or preparing meals [3,19], difficulty using kitchen items, poor coordination and visuospatial problems that impacted cooking safety, and swallowing or dental problems that increased aspiration risk while eating [3,19,20]. Functional challenges led to practical challenges including safety concerns for food shopping and preparation, increased care needs and role changes, and nutritional intake concerns for persons living with ADRDs [3,20,21]. As a result, persons living with dementia described experiencing increased social isolation, reluctance to receive support from caregivers, and resistance to changing cherished parts of mealtime routines [19,22]. For caregivers, the functional and practical challenges associated with mealtimes led to insecurity about cooking abilities and preparing nutritional meals, anxiety surrounding how to best support persons living with dementia during mealtimes, and intense emotions such as guilt, shame, and overwhelm [[21], [22], [23], [24]]. Over time, these compounding challenges also led to strained relationships between the caregiver and care recipient [[21], [22], [23], [24]].

### 3.5 Coping strategies and potential interventions

Several studies described the benefits of cognitive rehabilitation strategies (e.g., simplifying tasks, using visual and written cues, decluttering environment and minimizing distractions, using assistive devices, using color contrast dishware) and ongoing safety assessments for persons living with dementia to address functional challenges with meal preparation (food shopping, cooking, and related tasks) [3,25]. Many studies also suggested changes to routines like eating together as a family, allowing more time for meals, minimizing distractions during meals, preparing familiar meals at routine times (e.g., soup at dinner), engaging in meaningful conversation, and occasionally eating at restaurants or ordering out to reduce stress of preparing meals [13,19,23,[25], [26], [27]]. For caregivers, studies emphasized the benefits of: cultivating patience, flexibility, and creative thinking [19,26]; honoring the care recipient’s identity and treating them with dignity [26,27]; taking breaks during mealtime routines [28]; and tailoring support to their loved ones’ preferences [25]. Studies also emphasized the importance of individuals and caregivers building tolerance for errors or persistent challenges, learning to communicate openly about support preferences and needs, cultivating acceptance and flexibility in the face of challenges, and finding meaning in shared mealtime experiences [19,22,28].

### 3.6 Preferred outcomes

Only a few studies provided information on the preferred outcomes of persons living with dementia and caregivers as it pertained to food-related interventions. One study highlighted the importance of reducing feeding difficulties such as low food intake, unintentional weight loss, and anxiety surrounding food preparation and meals [19]. Another study suggested focusing on caregivers’ readiness for managing support needs and easing transitions for persons living with dementia to be helpful in improving caregiver competence and emotional wellbeing [22]. Two studies emphasized the importance of mealtime enhancement and improving dyadic interactions, with the goal of reducing challenges and making mealtime experiences more predictable and pleasurable [13,23].

### 3.7 Intervention characteristics (procedures, format, content)

Table 2 provides a detailed summary of intervention studies (n = 10), including the intervention procedures, format, and content. Most studies were quantitative, qualitative, or mixed methods descriptive evaluations of user impressions (n = 5), followed by randomized clinical or controlled trials (n = 3), and a nonrandomized open pilot (n = 2). Several interventions provided cognitive rehabilitation and cooking training (n = 3), followed by general instruction and support to increase knowledge and participation in daily activities, with some focus on cooking and mealtime engagement (n = 3). Others developed educational pamphlets or programs (n = 2), and some studies were focused on various forms of assistance for mealtimes such as meal delivery programs (n = 1) or assistive devices (n = 1). Most interventions were focused on persons living with dementia and caregivers (n = 4) or persons living with dementia only (n = 4), and some focusing on caregivers only (n = 2).

**Table 2:** Summary of findings from intervention studies (n = 10).

Reference	Study Aim	Study Design/ Participant Characteristics	Relevant Findings	
			Intervention Characteristics (procedures, format, content)	Reported benefits
1.Bayne 2024	To investigate the impact of targeted, individualized caregiver training via telehealth with the teach-back method on mealtime management on caregiver outcomes	Dissertation study. Mixed methods single case analyses of caregivers at two time points **Caregivers (N = 3)** Sex: Female n = 2 Male n = 1 Race: Black n = 2 White n = 1 Relationship: Child n = 2 Nephew n = 1	Procedures: Caregivers participated in an intake interview that was used to create an education program, and then participated in a multicomponent educational session, which could have included: 1) increased sensory stimulation, 2) social environment manipulation, 3) consideration of care recipient’s preferences, 4) increased cognitive stimulation, and 5) ensure appropriate motor support. Format: One 30-90 min online (Zoom) educational session including education and suggestions for modification. Content: Based on the biopsychosocial model of mealtimes for persons with dementia (Bayne & Shune, 2022)	Quantitative findings: - caregivers exhibited significant increases in quality of life and confidence -care recipient’s food intake increased or stabilized for all participants
2. Brandao et al., 2022	To examine the feasibility of an intergenerational remote intervention program designed to promote the wellbeing and social connection of vulnerable older adults, including people with dementia	Mixed methods study involving quantitative descriptive analysis (pre-post program) and qualitative synthesis of participant observations **Persons living with dementia (n = 5)** *No demographic information reported on subgroup living with dementia	Procedures: Undergraduate students were guided to lead weekly sessions of clowning, storytelling, dancing, and cooking‐related activities for 3 months. Format: Weekly 60 min sessions over Zoom, with messaging over WhatsApp Content: Discussion of themes including communication, memory, and imagination for autonomous activities including cooking	Quantitative findings: -Feasibility- High enrollment of those invited (81%); and high retention (77%) of those enrolled; plus medium/high attendance by most (83.7%) participants Qualitative Findings: -evaluative conversation sessions at the end of the 3‐month period showed that participants perceived the program as supportive and reported feelings of belonging. - in all groups, the most frequent answers of participants were positive assertions on social connection
3. Curtin, 2011	To explore the effectiveness of a nurse-led individualized skills training (IST) program for individuals with mild Alzheimer’s Disease in the home setting.	Non-randomized open pilot trial with qualitative analysis using content analysis **Clients with Alzheimer’s disease and caregivers (N = 5 dyads) Persons living with dementia (n = 5):** Sex: Female (n = 5) Age: 71−85 Education: 8−14 years Race/ethnicity: Caucasian (n = 5) **Caregivers: (n = 5)** Relationship: Husband (n = 3) Adult child (n = 1) Hired aid (n = 1)	Procedures: 5 60-minute training sessions involving observation and a structured, repetitive teaching approach using verbal, visual, and physical prompting for a routine mealtime task (preparing a breakfast meal) Format: Direct observation in a home-based program; followed by 5 training sessions with a nurse and 5 “maintenance” sessions with a caregiver Content: Verbal, visual, physical prompting during mealtime preparation	Qualitative Findings: -clients with mild Alzheimer’s disease (AD) who were all previously dependent on caregivers for meal preparation learned or relearned task of preparing breakfast -all showed improvement in independent preparation of meals; could make foods (e.g., pudding, pancakes, poached eggs, toast, muffins, oatmeal) with minimal verbal prompting -No clients achieved complete independence in the task. Qualitative: Key facilitators: supportive caregiver, participant’s insight, researcher’s presence, and individualized training (caregiver support was most important). Obstacles: overhelping or distraction by caregivers, participants, or researcher; improved as caregivers learned to give verbal, not physical, prompts. Perceptions: caregivers enjoyed the program, saw greater participant confidence and independence, and valued the researcher’s calming, supportive role. The program validated caregivers’ methods and encouraged a shift from doing tasks for participants to promoting independence.
4. Murai & Yamaguchi, 2017	To evaluate the effects of a cooking program as cognitive rehabilitation for elderly individuals with dementia.	Randomized controlled trial with quantitative outcome assessment before and after (3 months) condition completion **Persons living with dementia (N = 36)**; randomized to intervention (n = 18) or control (n = 18) conditions Age: Intervention: 86.1 +- 5.8 Control: 84.7+-7.2 Sex: Intervention: Male (n = 3) Female (n = 15) Control: Male (n = 4) Female (n = 14)	Procedures: Cooking program with varying weekly menu items, compared to a control condition that included participation in recreation (e.g., balloon volleyball, radio gymnastics, chorus) with care workers Format: 12 weekly sessions (90-minute) in small groups led by an occupational therapist Content: program included a menu of 12 Japanese style home-cooked dishes familiar to elderly people; based on five principles of brain-activating rehabilitation (BAR): fostering a pleasant atmosphere, interactive communication, establishing social roles, giving and receiving praise, and errorless learning.	Quantitative Findings: - no significant differences in groups at baseline -intervention group had high attendance (86.6%); and care staff reported positive impressions of program -cooking program significantly improved in executive function; declined in the control group -no significant differences in groups in mood symptoms, quality of life, activities of daily living -intervention effects were most notable for people with mild to moderate dementia; those with severe dementia showed little change
5. Nomura et al., 2009	To describe the implementation and evaluation of a program based on cognitive rehabilitation aimed at empowering the elderly with early dementia and education and counselling program aimed at likewise empowering their family caregivers.	Qualitative study of implementation and impressions of cognitive rehabilitation program obtained through focus groups using a participatory action research (PAR) model that lasted over 5 years **Persons living with dementia (N = 37)** Age: 78.9 + 6 Sex: Male (n = 11) Female (n = 26) Living situation: Alone (n = 9) Spouse (n = 10) Extended family (n = 15) Others (n = 3) **Family caregivers (N = 31)** Sex: Male (n = 9) Female (n = 22)	Procedures: programs integrated within community health model employed by community health nurses; participants recruited following needs-assessment survey and town campaign to improve health behaviors Format: 1) monthly in person group for persons living with dementia; 2) in person/ phone counseling on a monthly basis for caregivers Content: 1) For people living with dementia, included a monthly activity-based program focused on improving procedural skills and health knowledge in the morning, with other activities in the afternoon); 2) For caregivers, in-person and phone counseling from social workers and care managers focused on commending caregivers’ efforts, identifying problems and problem solving, and exploring strategies to manage care recipients’ behavioral symptoms.	Qualitative findings: -highlighted three stages of empowerment: individual, group, and community -participants believed that cognitive rehabilitation can restore lost procedural skills, and help regain self-confidence -positive feedback was important element in restoring caregiving confidence as well as education and counseling
6. Nygård et al., 2008	To identify the characteristics of elderly, urban stove-timer users with age-related memory deficits or dementia compared to those with other disabilities, and to examine the application, implementation, and timer-option procedures used in a publicly funded home-modification system.	Quantitative cross-sectional descriptive study of people with diagnosed or suspected dementia and those with other diagnoses **Persons living with dementia (N = 788)** Age: 83.5 (43−104) Sex: Female n = 597 Male n = 197	Procedures: Persons living with dementia applied for stove timers through an agency for home modification Format: Older adults were provided with a stover timer controlled through existing knobs on the stove and installed by an electrician; could allow for time set to turn off stove; device could be used as a heat sensory; and could have an auditory alarm signal Content: N/A. Stover timer technology	Stover-timer usage challenges: -heavy reliance on professional assistance (87.2%) -insufficient recommendations for use and lack of adaptations (e.g., auditory alarms, oven connections) -people with dementia received fewer timer-setting recommendations; lack of tailoring to user needs
7. Papachristou et al., 2018	To develop and assess the content, format, and usefulness of two booklets (one newly developed and one existing) from the perspectives of caregivers of people with dementia.	Qualitative thematic analysis of a booklet (“Food for thought; booklet 1) and a revised booklet (booklet 2) for caregivers focused on food-related processes **Caregivers of persons living with dementia (N = 20),** recruited in two phases (n = 10 in each phase) Age: 70 years old on average Sex: Female (n = 8) Male (n = 12) Relationship: Spouse/partner (n = 13) Adult child (n = 3) Grandchild (n = 2) Friend (n = 1)	Procedures: Participants randomly assigned to review booklet 1 or 2; think-aloud sessions conducted in participants homes with an experienced qualitative researcher Format: Two print copies with topics related to mealtimes and eating relevant for those navigating dementia Content: Both booklets contained information about eating and mealtime considerations for dementia (e.g., taste/ appetite changes, maintaining independence, mealtime routines, balancing support needs); booklet 2 contained enhanced information including food shopping, preparation, and eating	Qualitative findings: - caregivers resonated with advice on kitchen safety, weight maintenance, appetite changes, decision-making difficulties, hydration, and mealtime strategies - eating out seen as both challenging and manageable with preparation - format (clear layout, visuals, caregiver quotes) provided reassurance and normalization - booklets were generally useful and practical, though some advice for late-stage dementia felt daunting to those caring for earlier stages -booklet1, caregivers appreciated inclusion of challenges with dental and hygiene care -booklet 2 benefits included emphasis on addressing shopping challenges (e.g., not purchasing, overstocking, difficulties during outings), and maintaining shopping routines viewed as more widely applicable - both booklets were seen as credible, supportive, and aligned with caregivers lived experiences
8. Rapaport et al., 2021	To examine the feasibility and acceptability of NIDUS-Family, a 6–8 session manualized, individually tailored, modular intervention supporting independence at home for people with dementia; and explore participants’ and facilitators’ experiences of the intervention.	Multisite single arm open pilot trial with qualitative methods of people with dementia and carers **Persons with dementia (N = 15):** Age: 84.3 (14.2) Sex: Female (n = 8) Male (n = 7) Race/ethnicity: Black (n = 2) White (n = 12) Other (n = 1) **Caregivers (N = 15):** Age: 61.3 (14.1) Sex: Female (n = 10) Male (n = 5) Race/ethnicity: Black (n = 2) White (n = 13)	Procedures: NIDUS-Family intervention that allowed family carers and/or people living with dementia to select personal goals and modules to help them achieve goals Format: 6-8 sessions, delivered either to family carers alone or with person with dementia as a dyad Content: facilitators explored goals and mapped support networks, and then selected modules based on goals (e.g., making decisions, behaviors and emotions, physical activity, diet and healthy eating, difficulties eating and drinking, living well at home)	Qualitative findings: -personalized goal setting challenging but valuable; strict adherence to manual difficult -balancing needs of carers and people with dementia important; difficult to ensure carers needs not overlooked Quantitative findings: -program generally viewed as acceptable (75%) -fidelity to intervention high (81.5%) -goal attainment indicated that program exceeded baseline expectations
9. Thomas et al., 2023	To assess the risk of nursing home admission between homebound individuals receiving daily delivered vs drop-shipped frozen meals.	Pilot pragmatic randomized clinical trial with multiple sites (Texas and Florida) and longitudinal (6-month) assessment **Persons with Dementia (N = 243):** Age: 66−74 (n = 63) 75−84 (n = 96) >85 (n = 84) Sex: Female (n = 152) Male (n = 91) Race/ethnicity: Black (n = 43) Latino/Hispanic (n = 62) White (n = 128) Other (n = 10) Other information: 119 participants (49.0%) lived alone	Procedures: Participants randomized to either meals delivery by a meals on wheels (MOW) driver or had frozen meals delivered to their homes every 2 weeks; received assigned intervention for up to 6 months Format: 1) daily-delivered meals received 5 lunchtime meals to their home 2-5 times per week by a MOW program volunteer or driver, who also conducted an informal wellness check; 2) drop-shipped meals received 10 frozen meals mailed via Fedex to their home every 2 weeks from TRIO community meals Content: N/A. Food delivery intervention.	Quantitative (survival analyses): -participants in the daily-delivered meals arm were less likely to be admitted to a nursing home compared with the drop-shipped frozen meals arm, although the result was not significant (difference, 6.0 percentage points; 95% CI, −14.0 to 1.5 percentage points). −17 participants (7.1%; 95% CI, 3.9 %–10.4 %) had died. -program demonstrated high feasibility of enrollment, monitoring with fidelity, linking data, measuring outcomes
10. Yu et al., 2023	To examine whether a dyadic cooking-based intervention can improve the subjective health and well-being of older adults with SCD and their caregivers, as well as the cooking competence of the former group.	Randomized controlled trial of cooking intervention versus wait-list control **Adults with subjective cognitive decline (n = 60)** Age: 78.4 (8.8) Gender: Men (n = 27) Women (n = 33) **Caregivers (n = 60)** Age: 65.3 (11.8) Gender: Men (n = 12) Women (n = 48)	Procedures: Orientation session with occupational therapist and social worker, who introduced cognitive training using procedural learning paradigm and tips for relieving caregiver stress, then weekly cooking classes run by trained cooking instructor. Format: in person orientation session, followed by 5 weekly 2 -h cooking classes in a group format; cookbook included 5 Chinese recipes with step-by-step instructions Content: Intervention involved group-based nutrition education aiming to promote cooking safety, confidence, and skills, and create social network support and resources. Each cooking class included nutrition education, recipe cooking from a class cookbook, and meal sharing with other participants.	Quantitative findings: -both older adults with SCD and caregivers had increases in well-being that differed from control (β = 0.508, 95% CI [0.036, 0.980]) -older adults with SCD increased in cooking competence relative to control (β = 1.629, 95% CI [0.165, 3.071]) Qualitative findings: - high satisfaction with cooking classes and overall enjoyed program -found cookbook helpful and easy to use

#### 3.7.1 Cooking training with embedded cognitive rehabilitation

A non-randomized open pilot trial explored the impact of a nurse-led skills training program for individuals with mild Alzheimer’s disease and caregivers delivered at home (N = 5 dyads) [29]. The program included 5 training (nurse-led) and 5 observation (caregiver-led) sessions involving a teaching approach emphasizing repetition in preparing the same breakfast meals with verbal, visual, and physical prompting during the meal preparation. The program led to preliminary evidence of improvements for all persons living with dementia in terms of independent preparation of breakfast meals, though no participants achieved complete independence. A randomized controlled trial compared a 12-week small group cooking program with embedded cognitive rehabilitation to a control condition (recreational activities with care workers) for persons living with dementia (N = 36) and observed high feasibility and acceptability for the intervention condition, and significant improvements in executive function in the intervention condition relative to the control group [30]. Finally, one randomized controlled trial evaluated a dyadic cooking-based intervention with embedded cognitive training for those living with subjective cognitive decline and their caregivers (N = 60) relative to a wait-list control [8]. Dyads in the intervention condition had significant improvements in well-being, and older adults with subjective cognitive decline had significant increases in cooking competence relative to controls.

#### 3.7.2 Engagement in activities of daily living

One study gathered qualitative feedback from of persons living with dementia (N = 37) and their family caregivers (N = 31) about an established community-based program that focused on empowering persons living with dementia and caregivers [31]. The program offered monthly cognitive rehabilitation for persons living with dementia focused on improving procedural skills and health knowledge. Additionally, the program provided monthly counseling for caregivers focused on increasing support, problem solving, and exploring behavioral management strategies. Qualitative feedback indicated that cognitive rehabilitation was helpful to restore lost skills and build self-confidence for persons living with dementia, while positive feedback and education was helpful for caregivers. A non-randomized multisite feasibility trial examined a 6−8 session goal setting intervention (NIDUS-Family) with N = 15 dyads that included discussion of activity participation, meal preparation, and ways of managing behavioral symptoms. The intervention demonstrated high feasibility and acceptability ( >75 %) and participants indicated that they achieved their goals at a level that exceeded their initial expectations. Finally, one study employed undergraduate students to lead weekly 60-minute sessions of clowning, storytelling, dancing, and cooking-related activities to persons living with dementia (N = 5) for 3 months over Zoom and WhatsApp [32]. The program demonstrated high feasibility (enrollment, retention, attendance >77 %) and was viewed positively by participants for improving their feelings of social connectedness.

#### 3.7.3 Educational programs

A mixed methods study examined the impact of a targeted, individualized caregiver training based on the biopsychosocial model of mealtimes in a series of single case analyses of caregivers (N = 3). Caregivers participated in an intake interview and then a personalized multicomponent education session (60−90 min over Zoom) focused on recommendations for supporting care recipients [33]. Caregivers exhibited significant increases in quality of life and confidence, and care recipients’ food intake increased or remained stable for all participants. Another study described a qualitative analysis of caregivers’ (N = 20) perspectives on two booklets focused on food-related processes developed with qualitative feedback [34]. The study compared: (1) the original “Food for Thought,” booklet that included information on food-related processes such as maintaining mealtime routines, supporting independence, and navigating practical challenges, to (2) a revised booklet with additional information on shopping and meal preparation, plus an index section. Qualitative analyses revealed that caregivers found both booklets credible, supportive, and aligned with the caregivers’ lived experience.

#### 3.7.4 Meal delivery and assistive devices

One multisite randomized clinical trial compared daily delivered meals through a Meals on Wheels (MOW) program to a drop-shipped frozen home meal program among older adults with dementia (N = 243) and assessed 6-month risk of nursing home admission [35]. The trial demonstrated high feasibility of enrollment, monitoring with fidelity, linking data, and measuring outcomes, and participants in the MOW arm were less likely to be admitted to a nursing home compared with the drop-shipped frozen meals arm (difference, 6.0 percentage points; 95 % CI, −14.0–1.5 percentage points). A quantitative cross-sectional descriptive study examined the characteristics of a stove-timer application program tailored to persons diagnosed or suspected with dementia at home (N = 788). Older adults were provided with a stove timer controlled through existing knobs on the stove and installed by an electrician that allowed for customization. Results indicated that participants received insufficient recommendations for use and a lack of instructions on adaptations (e.g., auditory alarms, connection to ovens) that led to a heavy reliance on professional assistance (87.2 %), suggesting that greater tailoring to users’ needs would be important in future interventions.

## 4 Discussion

This scoping review had the primary objective of summarizing the available literature on cooking and mealtime interventions for individuals with early stage ADRDs and their caregivers in community settings to promote the future development, testing, and implementation of interventions. Our goal was to synthesize information that could be used to develop programs capable of improving nutritional intake, physical and emotional health, social engagement, and caregiver uncertainty and distress. Our review included 22 qualitative and intervention studies across 9 countries. Overall, our findings confirmed that this is an emerging area of research with some promising early evaluations of interventions that present promising avenues for future research.

Overall, cooking interventions with embedded cognitive rehabilitation had the strongest preliminary evidence of improving functional independence, well-being, and cooking competence for persons living with dementia. They had the added advantage of being conducted in randomized trials (evaluated against minimally enhanced usual care or waitlist control conditions [8,29,30]), and should be further tested to assess their efficacy in fully powered trials. Educational programs and meal delivery interventions also demonstrated early feasibility and acceptability [[33], [34], [35]], and could be suitable for further feasibility and efficacy testing.

Our review elucidated several potential “building blocks” for addressing the overlapping functional, practical and emotional challenges that persons living with dementia and caregivers experience surrounding mealtime routines. For overlapping functional challenges (e.g., difficulty recognizing food/food spoilage, using kitchen items, maintaining cooking safety, chewing or swallowing food) and practical challenges (e.g., safe food preparation, increased care needs, role changes, reduced nutritional intake), interventions could begin with multidisciplinary assessment (e.g., occupational therapists, nutritionists, registered dietitians, social workers/case management) to determine current capabilities and tailor suggestions. This assessment can be used to offer specific suggestions for environmental modification (e.g., using labels or visual cues in kitchen, dated food storage systems, written directions for food preparation, checklists for grocery shopping and food in kitchen). Additionally, qualitative studies in our review highlighted helpful strategies such as reducing distractions, creating routines for meal preparation and cooking, and allowing more time for meal preparation routines. Routines could be supported through evidence-based cognitive rehabilitation strategies, such as breaking down tasks into written step-by-step instructions, simplifying tasks, learning through repetition, cognitive stimulation, and improvement of procedural skills through repeated practice. A robust literature base demonstrates that cognitive stimulation, cognitive therapy, and cognitive rehabilitation therapies in a variety of formats (individual, group, technology-based) can improve cognitive functions for people with mild dementia [36], and such approaches should also be examined in connection to mealtime routine participation outcomes.

While no intervention studies specifically targeted emotional distress or communication challenges, their mention in several qualitative studies in our review [3,19,22,[25], [26], [27], [28]] suggest that tools to address such challenges could be important components of cooking and mealtime interventions. Specifically, mindfulness and relaxation training has demonstrated benefits on psychological flexibility, distress tolerance, and levels of emotional distress for individuals living with dementia and their caregivers [37]. In addition, training in problem solving and collaborative communication has also successfully improved outcomes for dyads navigating the early challenges of dementia [38], and could be integrated into cooking and mealtime interventions.

Our review also identified important constructs that can be assessed with validated questionnaires in prospective studies and intervention trials. The Mini Nutritional Assessment-Short Form, an 8-item assessment of food intake, unintentional weight loss, and mood, can serve as a screening measure to identify individuals that might benefit from cooking and mealtime interventions [39]. Regarding mealtime routine participation, self-report questionnaires such as the Cooking Competence (14-items) and Food Skills (19-items) measures can serve as brief needs assessments and help to tailor interventions [40]. Performance based measures like the Kitchen Task Assessment can also be used to identify specific cognitive challenges related to mealtime difficulties [41]. Additionally, studies can investigate whether increases in caregiver competence serve to benefit their own well-being, and the well-being of the person living with dementia, and could include measures such as the 3-item Caregiving Competence Scale [42] and the 5-item World Health Organization-Five Well-Being Index [43] Finally, future studies should assess the impact of cooking and mealtime interventions on relationship dynamics, including the quality of mealtimes using measures like the 10-item Family Dinner Quality Scale [44] and the 5-item Positive Affect Index [45] which assess for closeness, communication quality, similarity in views, and shared activities.

### 4.1 Limitations of the scoping review

Although we included studies from 9 countries, our review was limited to studies published in English, which may limit the transferability of findings globally. In addition, we did not formally assess the methodological quality of included studies given the nascent state of the available literature. While quality appraisal is not recommended in reporting guidelines for scoping reviews [16], our results highlighting potential intervention effects should be interpreted with caution. In addition, most studies were qualitative and included relatively small sample sizes; intervention studies that were included were small pilots and feasibility studies, and therefore appropriately did not include extended follow up or strategies for implementation. Future prospective studies and intervention studies with follow-up assessment are needed to understand the potential impact and scalability of cooking and mealtime strategies and interventions.

## 5 Conclusion

Our scoping review characterized the state of the literature surrounding cooking and mealtime interventions for persons with dementia and their caregivers in community settings. Our findings underscore the need for additional, more rigorous research focused on existing interventions, and the development of additional mechanism-driven interventions that incorporate the perspectives of intended users. Our findings can support the creation of such programs focused on individuals, family members and care dyads, and environmental modifications in home and community settings.

## CRediT authorship contribution statement

Sarah M. Bannon, PhD: conceptualization, methodology, formal analysis, investigation, resources, data curation, writing-original draft, writing- reviewing & editing, supervision, project administration. Renata Elizalde Carrillo, BA: formal analysis, investigation, data curation, writing-original draft, writing- reviewing & editing, visualization. Sydney McCage, MA: formal analysis, investigation, data curation, writing-original draft, writing- reviewing & editing, visualization. Muhammad Hamza Shahab, MD: conceptualization, methodology, formal analysis, data curation. Grace O. Allison: conceptualization, methodology, formal analysis, data curation, writing-original draft. Aida Aminpour, MA, MPH: formal analysis, investigation, data curation, writing-original draft, writing- reviewing & editing. Carrie Levinson, MSLIS: methodology, software, formal analysis, resources, data curation. Alexander M. Presciutti, PhD: writing- original draft; writing-review & editing. Anne Fishel, PhD: conceptualization, writing-review & editing, supervision.

## Ethical statement

This research was determined to be exempt from Institutional Review Board (IRB) oversight by the Icahn School of Medicine at Mount Sinai because it involved the secondary analysis of existing published research studies.

## Funding

We have no funding to disclose.

## Data availability statement

Data analysis instruments are available in the Multimedia Appendices. The data sets generated and/or analyzed during the current study are available from the corresponding author upon reasonable request.

## Declaration of competing interest

We declare no relevant conflicts of interest.

## Footnotes

Appendix A Supplementary data associated with this article can be found, in the online version, at http://dx.doi.org/10.1016/j.jnha.2026.101009.
