# Supplementary material for: New horizons in cooking and mealtime interventions for mild cognitive impairment and early dementia: a scoping review
Source: J Nutr Health Aging. 2026 Oct 20;30(12):101009. doi: 10.1016/j.jnha.2026.101009 (PMC13697821; doi:10.1016/j.jnha.2026.101009)
Supplement: Supplementary file 1 [file mmc1.docx]

**Supplemental Table 1.** *Synthesis of Databases Searched, Key Words, and Search Strategies*

| **Database** | Embase Classic Embase (Last search 5/8/2025) | MEDLINE(R) and Epub Ahead of Print, In-Process, In-Data-Review & Other Non-Indexed Citations, Daily and Version (Last search 5/8/2025) | PsycInfo (Last search 5/8/2025) | CINAHL (Last search 5/9/2025) |
| --- | --- | --- | --- | --- |
| **Key Words and Search Strategy** | 1 Meal/  2 exp Food Processing/  3 (meal* or cooking or food prep*).tw,kf.  4 exp Caregiver/  5 (individual* or "care partner*" or "caregiver" or "care giver*" or dyad*).tw,kf.  6 exp Cognitive defect/  7 ("subjective cognitive decline" or "mild cognitive impairment" or "cognitive dysfunction" or Alzheimer* or dementia*).tw,kf.  8 1 or 2 or 3  9 4 or 5  10 6 or 7  11 8 and 9 and 10 | 1 exp Meals/  2 exp Food Handling/  3 (meal* or cooking or food prep*).tw,kf.  4 Caregivers/  5 (individual* or "care partner*" or "caregiver" or "care giver*" or dyad*).tw,kf.  6 exp Dementia/  7 exp cognition disorders/  8 ("subjective cognitive decline" or "mild cognitive impairment" or "cognitive dysfunction" or Alzheimer* or dementia*).tw,kf.  9 1 or 2 or 3  10 4 or 5  11 6 or 7 or 8  12 9 and 10 and 11 | 1 Mealtimes/ or Eating Behavior/  2 exp Food Preparation/  3 (meal* or cooking or food prep*).mp.  4 Caregivers/  5 (individual* or "care partner*" or "caregiver" or "care giver*" or dyad*).mp.  6 exp Neurocognitive Disorders/  7 ("subjective cognitive decline" or "mild cognitive impairment" or "cognitive dysfunction" or Alzheimer* or dementia*).mp.  8 1 or 2 or 3  9 4 or 5  10 6 or 7  11 8 and 9 and 10 | ((MH "Meals") OR (MH "Meal Preparation+") OR (meal* or cooking or food prep*)) AND ((MH "Caregivers") OR (individual* OR "care partner*" OR "caregiver" OR "care giver*" OR dyad*)) AND ((MH "Dementia+") OR (MH "Cognition Disorders+") OR ("Subjective cognitive decline" OR "mild cognitive impairment" OR "cognitive dysfunction" OR Alzheimer* OR dementia*)) |

**Supplemental Table 2.** *Data Extraction Template*

| **Item** | **Article Data** |
| --- | --- |
| Study ID/Title |  |
| Country |  |
| Study design |  |
| Aim/ Objective |  |
| Start/ End data collection |  |
| Types of participants included |  |
| Population description |  |
| Eligibility criteria |  |
| Method of recruitment |  |
| Total N participants |  |
| Methods used (qualitative, mixed methods, quantitative) |  |
| Data analysis description |  |
| Demographic characteristics |  |
| Quantitative outcomes |  |
| Qualitative outcomes |  |
| Discussion/Conclusion points |  |
| Notes |  |

**Supplemental Table 2.** *Characteristics of Included Studies (N=22)*

| **Reference** | **Country** | **Study Design** | | **Populations** | | | **Setting** |
| --- | --- | --- | --- | --- | --- | --- | --- |
|  |  | Qualitative/ Mixed Methods  n=12 | Intervention  n=10 | Persons living with dementia  n= 13 | Caregivers  n= 16 | Healthcare Professionals  n=3 |  |
| 1. Ball et al., 2015 | Australia | X |  |  | X |  | Community |
| 2. Bayne 2024 | United States |  | X (Dissertation; case analysis) | X | X |  | Community |
| 3. Brandao et al.,, 2022 | Brazil |  | X  (Qualitative descriptive) | X |  |  | Clinic and Community |
| 4. Curtin 2011 | United States |  | X  (Open Pilot) | X | X | X | Clinic |
| 5. Fjellström et al., 2010 | Sweden | X |  |  | X |  | Clinic |
| 6. Genoe et al., 2012 | Canada | X |  | X | X |  | Community |
| 7. Johansson et al., 2017 | Sweden | X |  |  |  | X | Clinic |
| 8. Juckett et al., 2024 | United States | X |  |  |  | X | Community |
| 9. Keller et al., 2007 | Canada | X |  |  | X |  | Community |
| 10. Keller et al., 2010 | Canada | X |  | X | X |  | Clinic and Community |
| 11. Keller et al., 2015 | Canada | X (Longitudinal) |  | X | X |  | Clinic and Community |
| 12. Lam & Keller, 2015 | Canada | X |  | X | X |  | Community |
| 13. Mole et al., 2021 | United Kingdom | X |  |  | X |  | Community |
| 14. Murai & Yamaguchi, 2017 | Japan |  | X  (RCT) | X |  |  | Clinic |
| 15. Nomura et al., 2009 | Japan |  | X  (Qualitative descriptive) | X | X |  | Community |
| 16. Nygård et al., 2008 | Sweden |  | X  (Cross-sectional descriptive) | X |  |  | Clinic |
| 17. Papachristou et al., 2013 | United Kingdom | X |  |  | X |  | Community |
| 18. Papachristou et al., 2018 | United Kingdom |  | X  (Qualitative descriptive) |  | X |  | Community |
| 19. Rapaport et al., 2021 | United Kingdom |  | X  (Open Pilot) | X | X |  | Clinic |
| 20.Sharma et al., 2024 | Malaysia | X |  |  | X |  | Community |
| 21. Thomas et al., 2023 | United States |  | X (RCT) | X |  |  | Community and Residential |
| 22.Yu et al., 2023 | Hong Kong |  | X (RCT) | X | X |  | Community |

*Note:* RCT= randomized clinical or controlled trial
